# Critical Period Plasticity as a Framework for Psychedelic-Assisted Psychotherapy

**DOI:** 10.3389/fnins.2021.710004

**Published:** 2021-09-20

**Authors:** Lauren Lepow, Hirofumi Morishita, Rachel Yehuda

**Affiliations:** ^1^Department of Psychiatry, Icahn School of Medicine Mount Sinai, New York, NY, United States; ^2^Department of Neuroscience, Icahn School of Medicine Mount Sinai, New York, NY, United States; ^3^Department of Ophthalmology, Icahn School of Medicine Mount Sinai, New York, NY, United States; ^4^Department of Psychiatry, James J. Peters Veterans Affairs Medical Center, Bronx, NY, United States

**Keywords:** critical period plasticity, neuroplasticity, visual system, psychedelics, biological psychiatry, psychotherapy

## Abstract

As psychedelic compounds gain traction in psychiatry, there is a need to consider the active mechanism to explain the effect observed in randomized clinical trials. Traditionally, biological psychiatry has asked how compounds affect the causal pathways of illness to reduce symptoms and therefore focus on analysis of the pharmacologic properties. In psychedelic-assisted psychotherapy (PAP), there is debate about whether ingestion of the psychedelic alone is thought to be responsible for the clinical outcome. A question arises how the medication and psychotherapeutic intervention together might lead to neurobiological changes that underlie recovery from illness such as post-traumatic stress disorder (PTSD). This paper offers a framework for investigating the neurobiological basis of PAP by extrapolating from models used to explain how a pharmacologic intervention might create an optimal brain state during which environmental input has enduring effects. Specifically, there are developmental “critical” periods (CP) with exquisite sensitivity to environmental input; the biological characteristics are largely unknown. We discuss a hypothesis that psychedelics may remove the brakes on adult neuroplasticity, inducing a state similar to that of neurodevelopment. In the visual system, progress has been made both in identifying the biological conditions which distinguishes the CP and in manipulating the active ingredients with the idea that we might pharmacologically reopen a critical period in adulthood. We highlight ocular dominance plasticity (ODP) in the visual system as a model for characterizing CP in limbic systems relevant to psychiatry. A CP framework may help to integrate the neuroscientific inquiry with the influence of the environment both in development and in PAP.

## Introduction

The psychedelic treatment paradigm is one in which, following a comprehensive preparation process, a psychedelic medicine is administered for one or up to several sessions. The psychedelic produces an altered state of consciousness which is thought to facilitate self-exploration and insight. These insights are then examined in integration sessions in the absence of the medicine (Doblin et al., 2019). This treatment approach is different from the prevailing model of psychopharmacology in which a medication is used to bring about symptom reduction by providing corrective neurochemical effects on faulty pathways implicated in psychiatric disorders (DePierro et al., 2019). There remains debate about the centrality of the psychotherapy and the acute subjective experience in the psychedelic treatment (Heifets and Malenka, 2019; Olson, 2021; Yaden and Griffiths, 2021). Moreover, it has been suggested that the therapeutic properties of the psychedelic may not require the altered state of consciousness, but is rather based on the pharmacologic profile of the compound (Olson, 2021). Consideration of the extra-pharmacologic factors challenges the classic translational approach of biological psychiatry based in psychopharmacology that targets and reverses causal pathways of disease (DePierro et al., 2019).

Indeed, a prevailing model born from translational research is that chronic stress causes pathological atrophy of cortical neurons in the prefrontal cortex which can be reversed by rapid-acting antidepressant compounds such as ketamine, thus abolishing the patient’s psychiatric symptoms (Duman et al., 2016; Moda-Sava et al., 2019). Some argue that psychedelics can be fit into this model since they are thought to engage familiar neuroplasticity targets such as the serotonin 5-hydroxytryptamine-2A receptor (5-HT2AR), the glutamatergic α-amino-3-hydroxy-5-methyl-4-isoxazolepropionic acid (AMPA) pathway, and brain-derived neurotrophic factor (BDNF)/tropomyosin receptor kinase B (TrkB) signaling in cortical regions (Kadriu et al., 2021). The neuroplastic effect is seen by induced *structural changes*: dendrites in cell culture (Ly et al., 2018); *functional changes*: increased mean firing rate of layer 5 pyramidal neurons in the prefrontal cortex (Vollenweider and Preller, 2020); and *epigenetic changes:* neuroplasticity-related genes (Martin and Nichols, 2018). Novel therapeutics that target these known pathways with increasing specificity and potency yet lack accompanying psychoactive/experiential effects are in the drug development pipeline (Cameron et al., 2021).

However, a different perspective from clinical researchers working with psychedelics has emphasized the centrality of a deep psychological or spiritual exploration that is induced by the compound when given in the appropriate context (Greer and Tolbert, 1986; Belser et al., 2017; Bogenschutz and Forcehimes, 2017; Sessa, 2017; Wagner et al., 2017; Feduccia and Mithoefer, 2018; Garcia-Romeu and Richards, 2018; Nielson and Guss, 2018; Breeksema et al., 2020; Reiff et al., 2020; Sloshower et al., 2020; Mithoefer, n.d.). The common recreational use of psychedelics, yet lack of spontaneous psychiatric illness remission, and even potential psychological harm, in users calls attention to the importance of extra-pharmacologic factors. Yet because of what data is currently available and currently possible to obtain, the two sides remain valid opposing theoretical viewpoints.

To integrate these seemingly disparate mechanisms of action—biological and psychological—one does not have to look far to find a wealth of literature that purports that neuroplasticity can provide a biological model of how the response to the environment manifests in neurobiological change. Highly cited articles such as Castrén (2005); Branchi (2011), and Karpova et al. (2011) have proposed variations of the “undirected susceptibility to change model” (Branchi, 2011) that the mechanism of antidepressants may be to enhance the malleability of relevant brain circuits to environmental influence. Therefore, the range of clinical outcomes that results from manipulating neural plasticity may greatly depend on the environmental context in which they are taken. Nonetheless, a decade later, induction of synaptic neuroplasticity is still viewed as a proxy or biomarker for positive clinical effect (Moda-Sava et al., 2019; Cameron et al., 2021). To make progress in understanding the neurobiology of psychedelic assisted psychotherapy (PAP), we suggest investigating the interaction of the psychopharmacology and its context. This requires adopting a new theoretical approach to design studies to help explain mechanism.

Now we turn to consider a developmental framework that advancement in psychiatric treatment may result from the discovery of interventions that “release the brakes that retard” adult neuroplasticity (McEwen, 2012) to induce the heightened sensitivity to the environment observed during specific periods of development. “Neuroplasticity” is a heterogeneous phenomenon that has become an imprecise term. This review addresses a specific type of *in vivo* neuroplasticity in living animals: critical period plasticity (CPP), as seen in ocular dominance plasticity (ODP) in the visual system.

A critical period is a window in which environmental input is necessary for the appropriate development of the relevant brain circuit. During a critical period, the brain has a heightened plasticity in which experiences have robust effects on establishing stable neurocircuitry. During this developmental period, the brain’s malleability creates both a vulnerability to environmental insults or deprivations as well as a remarkable ability to quickly and robustly acquire skills. After the closure of critical periods, neuronal changes are still possible, but more restricted. Recent studies using the ODP model indicate that the nature and the underlying mechanisms of this juvenile form of robust plasticity –CPP– are distinct from adult types of neuroplasticity (Hensch, 2005; Morishita and Hensch, 2008). While various studies have correlated critical periods to synaptic plasticity mechanisms (Feldman, 2000), other cellular processes have been increasingly implicated in causally regulating CPP (Nabel and Morishita, 2013). The set of mechanisms involved in the opening, maintenance, and closing of CPP in primary sensory modalities—and in ODP specifically—have been elucidated, thus its candidacy to serve as a model system.

This paper highlights advancements in visual science as a model system for elucidating the molecular and circuit machinery responsible for the opening and closing these sensitive periods of development. The previous literature on CPP and other psychotropic medications is briefly reviewed. Finally, a case is made for investigating PAP through the lens of CPP. Perhaps these biological underpinnings of CPP can help understand how encounters with salient environmental stimuli during a sensitive period of development or a psychedelic experience durably alter functional neurocircuitry.

Of note, the definition of a psychedelic in clinical psychiatry, in the community, and in neuroscience remains imprecise. For example, while ketamine shares pharmacologic properties with psychedelics, it also has distinctions (Kadriu et al., 2021). While the psychedelic-like nature of ketamine continues to be explored (Mathai et al., 2020), it has not been packaged together with psychotherapy for FDA approval as have 3,4-Methylenedioxymethamphetamine (MDMA) and psilocybin and therefore was not included here as a psychedelic.

## Sensitive Periods of Psychological Development

During development, sensitive periods occur in which the brain is particularly sensitive to environmental input (Knudsen, 2004). If an adverse experience or deprivation occurs during a psychological stage of development, it has the power to affect lifelong psychological functioning whereas experiencing the same events as an adult may not have as robust an impact (Lupien et al., 2009; Pratchett and Yehuda, 2011). In developmental psychology, critical (or “sensitive”) periods have been characterized by rodent studies (Curley and Champagne, 2016), and naturalistic human deprivation studies such as institutional rearing (Bick and Nelson, 2016; Nelson et al., 2019). Such research informs the basis of early-intervention programs such as for Autism (Landa and Kalb, 2012).

Development of the emotional brain likely consists of many different overlapping sensitive periods of higher-order functioning such as attachment, emotion regulation, and social cognition (Piekarski et al., 2017). Adversity differentially affects the developing brain during critical periods (Nelson and Gabard-Durnam, 2020). Targeted enrichment in developmental domains are most effective during well-characterized temporal windows of opportunity (Marín, 2016). Yet the neurobiological mechanisms surrounding the opening and closing of these critical periods have not been well elucidated. Accordingly, there is a need for a model system in which the brain is disproportionately affected by the environment during discrete developmental periods.

## Critical Period Plasticity in the Visual System

CPP has been well formulated in the visual system across species (Wiesel, 1982; Hensch and Quinlan, 2018). ODP in the primary visual cortex (V1)—necessary for the development of binocular vision—is the most extensively studied form of CPP. Ocular dominance is reflected in the representation of the left versus right eye inputs into striate ocular dominance columns in V1. Similar to social and emotional development, a lack of expected environmental input to the relevant system during childhood either by insult or deprivation results in lasting deficits (Wiesel, 1982; Hensch and Quinlan, 2018). If one eye is deprived of normal input (e.g., from cataracts, or experimental monocular deprivation) during the well-defined critical period, the deprived eye will have poor visual acuity due to a shift of neuronal spiking response in V1 in favor of the open eye; amblyopia (“lazy eye”) will result (Wiesel, 1982; Hensch and Quinlan, 2018). If the strong eye is patched, amblyopia can be reversed and vision restored, but only if during the critical period in late childhood. Similarly, deprivation outside the critical period does not cause amblyopia (Wiesel, 1982; Hensch and Quinlan, 2018). The temporal window of CPP corresponds with a period of rapid physical growth when the distance between the two eyes increases and thus the visual receptive fields are constantly changing. The mechanism of both the opening and closing of the visual critical period has largely been elucidated, thus its utility as a model system.

By identifying the molecular brakes that typically halt visual CPP, pharmacologic intervention has made it possible to remove the brakes, thus re-opening visual CPP (Morishita and Hensch, 2008; Bavelier et al., 2010). Under these conditions, if the proper environmental stimulus is provided, near-blindness from amblyopia can be reversed in rodent models. But this intervention was not found by the traditional translational approach whereby solely modulating druggable targets in amblyopia’s pathophysiology reverses the illness without being in concert with proper experience. Instead, a pharmacologic intervention creates the molecular conditions in V1 whereby the brakes of CPP are released, and a specific environmental input (that was lacking or polluted during a time when it was necessary) can exert abiding effects (Vetencourt et al., 2008; Sale et al., 2014).

The translatability of molecular targets involved in visual CPP to psychological conditions has already begun to be explored. For example, the excitatory/inhibitory balance of interneurons that shifts during critical periods may also be relevant in limbic circuits (Kuhlman et al., 2013; Murthy et al., 2019). Key features of ODP including perineuronal nets, myelin-related nogo receptor signaling, and Lynx family proteins– key features in ocular dominance CPP– have signs of translatability including their involvement in fear-memory in the hippocampus, amygdala, and prefrontal cortex (Nabel and Morishita, 2013).

## Critical Periods and Psychotropic Medications

The relevance of visual CPP to psychiatry has been a topic revisited by many (Bavelier et al., 2010; Hensch and Bilimoria, 2012; McEwen, 2012; Castreń, 2013; Nabel and Morishita, 2013). Certain psychotropic medications, before the recent interest in psychedelics, have been highlighted for their unexpected induction of juvenile-like neuroplasticity in the visual system. The growing body of literature is briefly reviewed in this section.

Chronic fluoxetine treatment was shown to reinstate ODP in the adult amblyopic rat (Vetencourt et al., 2008). While a functional intervention for amblyopia is typically only corrective in childhood but not in adulthood, in this experiment, reinstatement of juvenile-like plasticity allowed for complete recovery of vision when paired with the functional intervention (Vetencourt et al., 2008). The induction of plasticity was a result of serotonin-induced reduction in intracortical GABAergic inhibition and increased BDNF expression leading to a shift the intracortical inhibitory-excitatory balance (Vetencourt et al., 2008). Furthermore, the study of ketamine, which similarly reopens a critical period for ODP, has cast light on the reopening process as an neuregulin-1-dependent restoration of excitatory input onto parvalbumin cells, thus enhancing cortical inhibition (Grieco et al., 2020). Ketamine’s rapid effect on ODP has also been shown to be mediated through TrkB signaling (Casarotto et al., 2021). Using ketamine as a tool has helped to better characterize the molecular components involved in the opening and closing of ODP critical periods.

The generalizability of critical periods in the visual system to that of affective circuits is of great relevance should this framework hold promise for the development and adoption of neuropsychiatric therapeutics (Spolidoro et al., 2008; Nabel and Morishita, 2013; Sale et al., 2014). The fear extinction paradigm has been targeted for investigation because of its quantifiable outcome and translatability from animal models. Typically, an enduring loss of a conditioned fear memory is only possible during a juvenile critical period, but not in adult mice. Indeed, selective serotonin reuptake inhibitors (SSRIs) (Karpova et al., 2011) and ketamine (Ju et al., 2017) both lead to enduring loss of a conditioned fear memory when paired with a fear extinction paradigm. Furthermore, fluoxetine’s structural and functional effect on the basolateral amygdala was evidence that the reopening of a CP in the fear circuit mirrors that of ODP (Karpova et al., 2011). These are early signs that certain psychotropics may reopen select affective critical periods such as fear, and that the mechanism of reopening in these circuits may be similar to that of reopening critical periods of ODP. At the same time, evidence calls into questions that ketamine’s antidepressant effects are plasticity-dependent (Moda-Sava et al., 2019; Abdallah et al., 2020).

As introduced above, SSRIs and ketamine have both been evaluated in the framework of ODP whereby the medication makes a circuit malleable for experiential input, both necessary for an enduring change. However, novel treatments in psychopharmacology have not been conceived, designed, or implemented as an aid to environmental input until the recent development of PAP. The concept of ODP is missing from the psychedelic literature and discourse though its relevance is promising.

## Psychedelics and Critical Period Plasticity

In a CPP framework, the therapeutic mechanism of psychedelics would be understood as the pharmacological properties of psychedelics putting the brain in a CP “open state,” while the psychotherapeutic aspect might retrieve appropriate engrams, such as traumatic memories (Inserra, 2018) for modification—now in a context of safety. Furthermore, the specific type and intensity of psychological support framing the experience has been correlated with clinical and efficacy outcomes (Griffiths et al., 2018).

As discussed above, the developmental influence on fear extinction shares features with ODP (Nabel and Morishita, 2013) and fear-extinction has been discussed as a potential component of the psychotherapeutic process in MDMA-assisted psychotherapy (Feduccia and Mithoefer, 2018). That psychedelics may reopen a psychosocial CPP has been corroborated in a rodent study of MDMA (Nardou et al., 2019). A social critical period was defined in mice behaviorally as an age-dependent peak in social reward learning. Biologically, this period of development was characterized by a change in the magnitude of oxytocin-dependent long-term depression (LTD) of glutamatergic inputs to medium spiny neurons in the nucleus accumbens. Intraperitoneal administration of MDMA reopened the behavioral critical period by binding to the serotonin transporter (SERT) and triggering a cascade leading to a metaplastic upregulation of oxytocin receptors (OXTR) and reopening of the social CPP and LTD at excitatory synapses (Nardou et al., 2019). So far, there is no known connection between the visual system CPP and this social learning CPP, so further investigation is required to know how molecular machinery of CPP may vary in different neural circuits.

Notably, MDMA’s reinstating CPP only occurred in a social context and not in isolated animals (Nardou et al., 2019), providing more data to suggest that biological intervention can be context-dependent (Hartogsohn, 2016, 2017; Haijen et al., 2018).

The 5-HT2AR often defines psychedelics as a class of medications and may also provide a nascent avenue for linking psychedelics and CPP. The synaptic plasticity associated with both the “serotonergic” psychedelics such as psilocybin as well as with MDMA has been found to be dependent on 5-HT2AR signaling (Ly et al., 2018; Olson, 2018; Vollenweider and Preller, 2020) and recently 5-HT2AR been thought to be involved in key developmental periods (Carhart-Harris and Nutt, 2017). Although, there is now competing evidence that psychedelic-induced plasticity may have a mechanism independent of 5-HT2AR (Hesselgrave et al., 2021).

More studies are required to fully assess the contribution of CPP re-opening to the mechanism of PAP, particularly in humans. An empirical question remains regarding whether MDMA and other psychedelics reopen ocular dominance CPP as ketamine does (Grieco et al., 2020) and what the implications would be for both visual science and psychiatry. An avenue for future research might be translatable assays that can detect re-opening of CP in humans.

## Discussion

**FIGURE 1:**
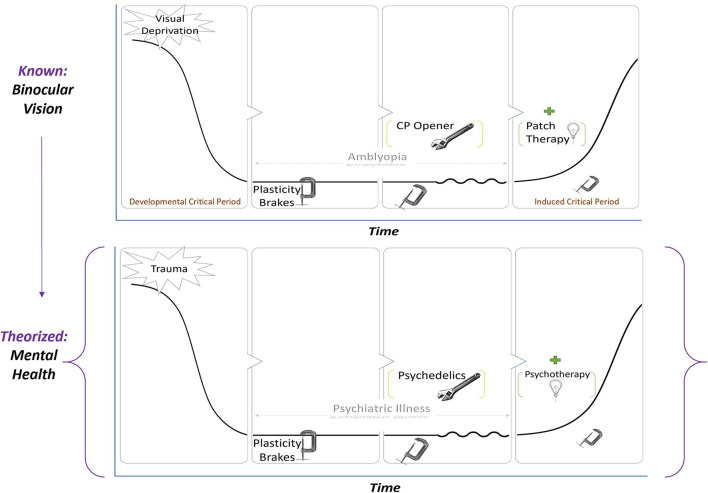
A known paradigm in visual CPP is applied to a hypothetical model of PAP. In both top and bottom leftmost panels, an adverse environmental input such as trauma, stress, or deprivation enduringly alters functioning, resulting in amblyopia (top) or psychiatric illness (bottom). Because of brakes on plasticity, there is a valley that restricts or prevents major recovery. In the third panel, the brakes are removed by critical period plasticity openers such as SSRIs and valproic acid in the case of vision or possibly psychedelics in the case of psychiatric illness. Removing the brakes alone does not restore functioning but rather makes the system sensitive to environmental input. A therapeutic intervention—patch therapy for vision and psychotherapy for mental health—during the period of induced plasticity is what returns the system to baseline.

The definition of CPP should be reiterated as a state in which neural networks are exquisitely sensitive to environmental inputs. Ocular dominance CPP may provide a theoretical framework (see Figure 1) for biological investigation of the synergistic effects of the psychopharmacologic and psychological properties of psychedelic-assisted-psychotherapy on clinical outcomes.

Many types and components of neuroplasticity (e.g., properties of a synapse, morphological plasticity, electrophysiological and biochemical changes, meta-plastic changes) are likely involved in psychedelics, but carefully characterizing and distinguishing the processes may have clinical implications. Not all neuroplasticity is therapeutic– for example, the morphological plasticity of cocaine is thought to be implicated in its abuse potential (Li et al., 2004; Ferrario et al., 2005; Russo et al., 2010; Marie et al., 2012), however (unlike MDMA), cocaine does not reopen a critical period for social reward learning (Nardou et al., 2019) in mice, illustrating just one instance where the distinctions between these “neuroplastic” processes may be therapeutically significant.

Building on a body of literature that calls attention to ocular dominance CPP as a potentially helpful framework for psychiatry, we posit its relevance to fine-tuning investigation of plasticity in psychedelic research. Moreover, ODP reflects physiological plasticity characterized in living organisms in contrast to other types of plasticity characterized *in vitro* or *ex vivo*. Therefore, CPP may orient future work to a different level of observation missing from psychedelic research. For example, the excitatory/inhibitory balance of interneurons, perineuronal nets, myelin-related nogo receptor signaling, and Lynx family proteins might be informative upstream biomarkers (Morishita and Hensch, 2008).

It is also possible that the mechanisms of ODP are not the most applicable to psychedelics. While the ODP critical period has been the most well-characterized, other critical periods—such as imprinting behavior in chicks (Jaynes, 1957), birdsong learning (Woolley and Rubel, 2002), rodent barrel cortex (Erzurumlu and Gaspar, 2012), post-stroke motor learning (Zeiler et al., 2013), cognitive flexibility (Gopnik et al., 2017), social/cognitive/affective domains in adolescence (Piekarski et al., 2017; Dahl et al., 2018), etc.—have been studied and likely do not share a single universal set of mechanisms. It seems unlikely that psychedelics might be a sort of master-key to reopen all types of critical periods even beyond the limbic system, but perhaps they might be a tool for better characterizing neuropsychiatric critical periods, as was begun with the critical period for social learning (Nardou et al., 2019). Furthermore, biomarkers associated with psychedelic-induced plasticity may shed light on the developmental biology of critical periods in limbic circuits (Castreń, 2013). However, a current lack of translational assays to measure changes in CPP-specific biomarkers limits the discussion to speculation. While some translational biomarkers of neuroplasticity have been developed (e.g., visually evoked potentials), they capture downstream observed changes in plasticity not specific to CPP (Normann et al., 2007).

If induction of CPP is a hypothesized mechanism for psychedelics, translational research studies will need to be re-evaluated to consider that what’s observed *in vitro* is only a part of the biological mechanism that explains the clinical effect. Equating neuroplastic-induction with clinical outcome misses an opportunity to rigorously investigate the many extra-pharmacologic factors that influence a patient’s clinical and biological outcome. Rather than stopping at trying to explain how a drug works, considering context may help our field with trying to explain what halts and what enables the brain to adapt to its environment. With psychedelics as a probe and the translational research paradigms of visual science to draw upon, perhaps biological psychiatry is better poised to understand and manipulate critical periods of plasticity in psychological development. Just as McEwen called himself a “molecular sociologist,” psychedelics may provide an opportunity to integrate neuroscientific inquiry with the psychosocial context in which a person heals.

## Data Availability Statement

The original contributions presented in the study are included in the article/Supplementary material, further inquiries can be directed to the corresponding author/s.

## Author Contributions

LL: preliminary concept guided and shaped by senior author, RY. HM: contribution of his expertise in the visual system and CPP. LL and RY: manuscript writing with edits by HM. HM: figure conceptualization, executed by LL and edited by RY. All authors contributed to the article and approved the submitted version.

## Conflict of Interest

The authors declare that the research was conducted in the absence of any commercial or financial relationships that could be construed as a potential conflict of interest.

## Publisher’s Note

All claims expressed in this article are solely those of the authors and do not necessarily represent those of their affiliated organizations, or those of the publisher, the editors and the reviewers. Any product that may be evaluated in this article, or claim that may be made by its manufacturer, is not guaranteed or endorsed by the publisher.

## References

[B1] Abdallah C. G. Averill L. A. Gueorguieva R. Goktas S. Purohit P. Ranganathan M. (2020). Modulation of the antidepressant effects of ketamine by the mTORC1 inhibitor rapamycin. *Neuropsychopharmacology* 45 990–997. 10.1038/s41386-020-0644-9 32092760 PMC7162891

[B2] Bavelier D. Levi D. M. Li R. W. Dan Y. Hensch T. K. (2010). Removing brakes on adult brain plasticity: from molecular to behavioral interventions. *J. Neurosci.* 30 14964–14971. 10.1523/JNEUROSCI.4812-10.2010 21068299 PMC2992973

[B3] Belser A. B. Agin-Liebes G. Swift T. C. Terrana S. Devenot N. Friedman H. L. (2017). Patient experiences of psilocybin-assisted psychotherapy: an interpretative phenomenological analysis. *J. Humanist. Psychol.* 57 354–388. 10.1177/0022167817706884

[B4] Bick J. Nelson C. A. (2016). Early adverse experiences and the developing brain. *Neuropsychopharmacology* 41 177–196. 10.1038/npp.2015.252 26334107 PMC4677140

[B5] Bogenschutz M. P. Forcehimes A. A. (2017). Development of a psychotherapeutic model for psilocybin-assisted treatment of alcoholism. *J. Humanist. Psychol*. 57 389–414. 10.1177/0022167816673493

[B6] Branchi I. (2011). The double edged sword of neural plasticity: increasing serotonin levels leads to both greater vulnerability to depression and improved capacity to recover. *Psychoneuroendocrinology* 36 339–351. 10.1016/j.psyneuen.2010.08.011 20875703

[B7] Breeksema J. J. Niemeijer A. R. Krediet E. Vermetten E. Schoevers R. A. (2020). Psychedelic treatments for psychiatric disorders: a systematic review and thematic synthesis of patient experiences in qualitative studies. *CNS drugs* 34 925–946. 10.1007/s40263-020-00748-y 32803732 PMC7447679

[B8] Cameron L. P. Tombari R. J. Lu J. Pell A. J. Hurley Z. Q. Ehinger Y. (2021). A non-hallucinogenic psychedelic analogue with therapeutic potential. *Nature* 589 474–479. 10.1038/s41586-020-3008-z 33299186 PMC7874389

[B9] Carhart-Harris R. L. Nutt D. J. (2017). Serotonin and brain function: a tale of two receptors. *J. Psychopharmacol.* 31 1091–1120. 10.1177/0269881117725915 28858536 PMC5606297

[B10] Casarotto P. C. Girych M. Fred S. M. Kovaleva V. Moliner R. Enkavi G. (2021). Antidepressant drugs act by directly binding to TRKB neurotrophin receptors. *Cell* 184 1299–1313.e19. 10.1016/J.CELL.2021.01.034 33606976 PMC7938888

[B11] Castreń E. (2013). Neuronal network plasticity and recovery from depression. *JAMA Psychiatry* 70 983–989. 10.1001/jamapsychiatry.2013.1 23842648

[B12] Castrén E. (2005). Is mood chemistry? *Nat. Rev. Neurosci.* 6 241–246. 10.1038/nrn1629 15738959

[B13] Curley J. P. Champagne F. A. (2016). Influence of maternal care on the developing brain: mechanisms, temporal dynamics and sensitive periods. *Front. Neuroendocrinol.* 40:52–66. 10.1016/j.yfrne.2015.11.001 26616341 PMC4783284

[B14] Dahl R. E. Allen N. B. Wilbrecht L. Suleiman A. B. (2018). Importance of investing in adolescence from a developmental science perspective. *Nature* 554 441–450. 10.1038/nature25770 29469094

[B15] DePierro J. Lepow L. Feder A. Yehuda R. (2019). Translating molecular and neuroendocrine findings in posttraumatic stress disorder and resilience to novel therapies. *Bio. Psychiatry* 86 454–463. 10.1016/j.biopsych.2019.07.009 31466562 PMC6907400

[B16] Doblin R. E. Christiansen M. Jerome L. Burge B. (2019). The past and future of science: an introduction to this Issue. *J. Psychoactive Drugs* 51 93–97. 10.1080/02791072.2019.1606472 31132970

[B17] Duman R. S. Aghajanian G. K. Sanacora G. Krystal J. H. (2016). Synaptic plasticity and depression: new insights from stress and rapid-acting antidepressants. *Nat. Med.* 22 238–249. 10.1038/nm.4050 26937618 PMC5405628

[B18] Erzurumlu R. S. Gaspar P. (2012). Development and critical period plasticity of the barrel cortex. *Eur. J. Neurosci.* 35 1540–1553. 10.1111/J.1460-9568.2012.08075.X 22607000 PMC3359866

[B19] Feduccia A. A. Mithoefer M. C. (2018). MDMA-assisted psychotherapy for PTSD: are memory reconsolidation and fear extinction underlying mechanisms? *Prog. Neuro Psychopharmacol. Bio. Psychiatry* 84 221–228. 10.1016/j.pnpbp.2018.03.003 29524515

[B20] Feldman D. E. (2000). Inhibition and plasticity. *Nat. Neurosci.* 3 303–304. 10.1038/73849 10725911

[B21] Ferrario C. R. Gorny G. Crombag H. S. Li Y. Kolb B. Robinson T. E. (2005). Neural and behavioral plasticity associated with the transition from controlled to escalated cocaine use. *Biol. Psychiatry* 58 751–759. 10.1016/j.biopsych.2005.04.046 16098484

[B22] Garcia-Romeu A. Richards W. A. (2018). Current perspectives on psychedelic therapy: use of serotonergic hallucinogens in clinical interventions. *Int. Rev. Psychiatry* 30 291–316. 10.1080/09540261.2018.1486289 30422079

[B23] Gopnik A. O’Grady S. Lucas C. G. Griffiths T. L. Wente A. Bridgers S. (2017). Changes in cognitive flexibility and hypothesis search across human life history from childhood to adolescence to adulthood. *Proc. Natl. Acad. Sci.U.S.A.* 114 7892–7899. 10.1073/PNAS.1700811114 28739917 PMC5544286

[B24] Greer G. Tolbert R. (1986). Subjective reports of the effects of MDMA in a clinical setting. *J. Psychoactive Drugs* 18 319–327. 10.1080/02791072.1986.10472364 2880946

[B25] Grieco S. F. Qiao X. Zheng X. Liu Y. Chen L. Zhang H. (2020). Subanesthetic ketamine reactivates adult cortical plasticity to restore vision from amblyopia. *Curr. Bio.* 30 3591–3603.e8. 10.1016/j.cub.2020.07.008 32822611 PMC7925140

[B26] Griffiths R. R. Johnson M. W. Richards W. A. Richards B. D. Jesse R. MacLean K. A. (2018). Psilocybin-occasioned mystical-type experience in combination with meditation and other spiritual practices produces enduring positive changes in psychological functioning and in trait measures of prosocial attitudes and behaviors. *J. Psychopharmacol.* 32 49–69. 10.1177/0269881117731279 29020861 PMC5772431

[B27] Haijen E. C. H. M. Kaelen M. Roseman L. Timmermann C. Kettner H. Russ S. (2018). Predicting responses to psychedelics: a prospective study. *Front. Pharmacol.* 9:897. 10.3389/fphar.2018.00897 30450045 PMC6225734

[B28] Hartogsohn I. (2016). Set and setting, psychedelics and the placebo response: an extra-pharmacological perspective on psychopharmacology. *J. Psychopharmacol*. 30 1259–1267. 10.1177/0269881116677852 27852960

[B29] Hartogsohn I. (2017). Constructing drug effects: a history of set and setting. *Drug Sci. Policy Law* 3:205032451668332. 10.1177/2050324516683325

[B30] Heifets B. D. Malenka R. C. (2019). Disruptive psychopharmacology. *JAMA Psychiatry* 76 775–776. 10.1001/jamapsychiatry.2019.1145 31241740 PMC8101021

[B31] Hensch T. K. (2005). Critical period plasticity in local cortical circuits. *Nat. Rev. Neurosci.* 6 877–888. 10.1038/nrn1787 16261181

[B32] Hensch T. K. Bilimoria P. M. (2012). Re-opening windows: manipulating critical periods for brain development.: the dana forum on brain science. *Cerebrum* 2012:11. PMC3574806 23447797

[B33] Hensch T. K. Quinlan E. M. (2018). Critical periods in amblyopia. *Vis. Neurosci.* 35:E014. 10.1017/S0952523817000219 29905116 PMC6047524

[B34] Hesselgrave N. Troppoli T. A. Wulff A. B. Cole A. B. Thompson S. M. (2021). Harnessing psilocybin: antidepressant-like behavioral and synaptic actions of psilocybin are independent of 5-HT2R activation in mice. *Proc. Natl. Acad. Sci. U.S.A.* 118:e2022489118. 10.1073/pnas.2022489118 33850049 PMC8092378

[B35] Inserra A. (2018). Hypothesis: the psychedelic Ayahuasca Heals traumatic memories via a sigma 1 receptor-mediated epigenetic-mnemonic process. *Front. Pharmacol.* 9:330. 10.3389/fphar.2018.00330 29674970 PMC5895707

[B36] Jaynes J. (1957). Imprinting: the interaction of learned and innate behavior: II. the critical period. *J. Comp. Physiol. Psychol.* 50 6–10. 10.1037/H0044716 13406129

[B37] Ju L.-S. Yang J.-J. Lei L. Xia J.-Y. Luo D. Ji M.-H. (2017). The combination of long-term ketamine and extinction training contributes to fear erasure by Bdnf methylation. *Front. Cell. Neurosci.* 11:100. 10.3389/FNCEL.2017.00100 28473755 PMC5398013

[B38] Kadriu B. Greenwald M. Henter I. D. Gilbert J. R. Kraus C. Park L. T. (2021). Ketamine and serotonergic psychedelics: common mechanisms underlying the effects of rapid-acting antidepressants. *Int. J. Neuropsychopharmacol.* 24 8–21. 10.1093/ijnp/pyaa087 33252694 PMC7816692

[B39] Karpova N. N. Pickenhagen A. Lindholm J. Tiraboschi E. Kulesskaya N. Ágústsdóttir A. (2011). Fear erasure in mice requires synergy between antidepressant drugs and extinction training. *Science* 334 1731–1734. 10.1126/science.1214592 22194582 PMC3929964

[B40] Knudsen E. I. (2004). Sensitive periods in the development of the brain and behavior. *J. Cogn. Neurosci.* 16 1412–1425. 10.1162/0898929042304796 15509387

[B41] Kuhlman S. J. Olivas N. D. Tring E. Ikrar T. Xu X. Trachtenberg J. T. (2013). A disinhibitory microcircuit initiates critical-period plasticity in the visual cortex. *Nature* 501 543–546. 10.1038/nature12485 23975100 PMC3962838

[B42] Landa R. J. Kalb L. G. (2012). Long-term outcomes of toddlers with autism spectrum disorders exposed to short-term intervention. *Pediatrics* 130 S186–S190. 10.1542/peds.2012-0900Q 23118250

[B43] Li Y. Acerbo M. J. Robinson T. E. (2004). The induction of behavioural sensitization is associated with cocaine-induced structural plasticity in the core (but not shell) of the nucleus accumbens. *Eur. J. Neurosci.* 20 1647–1654. 10.1111/j.1460-9568.2004.03612.x 15355332

[B44] Lupien S. J. McEwen B. S. Gunnar M. R. Heim C. (2009). Effects of stress throughout the lifespan on the brain, behaviour and cognition. *Nat. Rev. Neurosci.* 10 434–445. 10.1038/nrn2639 19401723

[B45] Ly C. Greb A. C. Cameron L. P. Wong J. M. Barragan E. V. Wilson P. C. (2018). Psychedelics promote structural and functional neural plasticity. *Cell Rep.* 23 3170–3182. 10.1016/j.celrep.2018.05.022 29898390 PMC6082376

[B46] Marie N. Canestrelli C. Noble F. (2012). Transfer of neuroplasticity from nucleus accumbens core to shell is required for cocaine reward. *PLoS One* 7. 10.1371/journal.pone.0030241 22272316 PMC3260254

[B47] Marín O. (2016). Developmental timing and critical windows for the treatment of psychiatric disorders. *Nat. Med.* 22 1229–1238. 10.1038/nm.4225 27783067

[B48] Martin D. A. Nichols C. D. (2018). The effects of hallucinogens on gene expression. *Curr. Top. Behav. Neurosci.* 36 137–158. 10.1007/7854_2017_479 28677095

[B49] Mathai D. S. Meyer M. J. Storch E. A. Kosten T. R. (2020). The relationship between subjective effects induced by a single dose of ketamine and treatment response in patients with major depressive disorder: a systematic review. *J. Affect. Disord*. 264 123–129. 10.1016/J.JAD.2019.12.023 32056741

[B50] McEwen B. S. (2012). Brain on stress: how the social environment gets under the skin. *Proc. Natl. Acad. Sci. U.S.A.* 109(Suppl. 2) 17180–17185. 10.1073/pnas.1121254109 23045648 PMC3477378

[B51] Mithoefer M. C. Mithoefer A. Jerome L. Ruse J. Gibson E. (n.d.). *A Manual for MDMA-Assisted Psychotherapy in the Treatment of Posttraumatic Stress Disorder.* Available online at: www.maps.org (accessed June 26, 2021).

[B52] Moda-Sava R. N. Murdock M. H. Parekh P. K. Fetcho R. N. Huang B. S. Huynh T. N. (2019). Sustained rescue of prefrontal circuit dysfunction by antidepressant-induced spine formation. *Science* 364:8078. 10.1126/SCIENCE.AAT8078 30975859 PMC6785189

[B53] Morishita H. Hensch T. K. (2008). Critical period revisited: impact on vision. *Curr. Opin. Neurobiol.* 18 101–107. 10.1016/j.conb.2008.05.009 18534841

[B54] Murthy S. Kane G. A. Katchur N. J. Lara Mejia P. S. Obiofuma G. Buschman T. J. (2019). Perineuronal nets, inhibitory interneurons, and anxiety-related ventral hippocampal neuronal oscillations are altered by early life adversity. *Biol. Psychiatry* 85 1011–1020. 10.1016/j.biopsych.2019.02.021 31027646 PMC6590696

[B55] Nabel E. M. Morishita H. (2013). Regulating critical period plasticity: insight from the visual system to fear circuitry for therapeutic interventions. *Front. Psychiatry* 4:146. 10.3389/fpsyt.2013.00146 24273519 PMC3822369

[B56] Nardou R. Lewis E. M. Rothhaas R. Xu R. Yang A. Boyden E. (2019). Oxytocin-dependent reopening of a social reward learning critical period with MDMA. *Nature* 569 116–120. 10.1038/s41586-019-1075-9 30944474

[B57] Nelson C. A. Gabard-Durnam L. J. (2020). Early adversity and critical periods: neurodevelopmental consequences of iolating the expectable environment. *Trends Neurosci.* 43 133–143. 10.1016/j.tins.2020.01.002 32101708 PMC8092448

[B58] Nelson C. A. Zeanah C. H. Fox N. A. (2019). How early experience shapes human development: the case of psychosocial deprivation. *Neural Plast*. 2019:1676285. 10.1155/2019/1676285 30774652 PMC6350537

[B59] Nielson E. M. Guss J. (2018). The influence of therapists’ first-hand experience with psychedelics on psychedelic-assisted psychotherapy research and therapist training. *J. Psychedelic Stud.* 2 64–73. 10.1556/2054.2018.009

[B60] Normann C. Schmitz D. Fürmaier A. Döing C. Bach M. (2007). long-term plasticity of visually evoked potentials in humans is altered in major depression. *Biol. Psychiatry* 62 373–380. 10.1016/J.BIOPSYCH.2006.10.006 17240361

[B61] Olson D. E. (2018). Psychoplastogens: a promising class of plasticity-promoting neurotherapeutics. *J. Exp. Neurosci.* 12:1179069518800508. 10.1177/1179069518800508 30262987 PMC6149016

[B62] Olson D. E. (2021). The subjective effects of psychedelics may not be necessary for their enduring therapeutic effects. *ACS Pharmacol. Transl. Sci.* 4 563–567. 10.1021/acsptsci.0c00192 33861218 PMC8033607

[B63] Piekarski D. J. Johnson C. M. Boivin J. R. Thomas A. W. Lin W. C. Delevich K. (2017). Does puberty mark a transition in sensitive periods for plasticity in the associative neocortex? *Brain Res.* 1654 123–144. 10.1016/j.brainres.2016.08.042 27590721 PMC5283387

[B64] Pratchett L. C. Yehuda R. (2011). Foundations of posttraumatic stress disorder: does early life trauma lead to adult posttraumatic stress disorder? *Dev. Psychopathol.* 23 477–491. 10.1017/S0954579411000186 23786690

[B65] Reiff C. M. Richman E. E. Nemeroff C. B. Carpenter L. L. Widge A. S. Rodriguez C. I. (2020). Psychedelics and psychedelic-assisted psychotherapy. *Am. J. Psychiatry* 177 391–410. 10.1176/appi.ajp.2019.19010035 32098487

[B66] Russo S. J. Dietz D. M. Dumitriu D. Morrison J. H. Malenka R. C. Nestler E. J. (2010). The addicted synapse: mechanisms of synaptic and structural plasticity in nucleus accumbens. *Trends Neurosci.* 33 267–276. 10.1016/j.tins.2010.02.002 20207024 PMC2891948

[B67] Sale A. Berardi N. Maffei L. (2014). Environment and brain plasticity: towards an endogenous pharmacotherapy. *Physiol. Rev.* 94 189–234. 10.1152/physrev.00036.2012 24382886

[B68] Sessa B. (2017). MDMA and PTSD treatment: “PTSD: from novel pathophysiology to innovative therapeutics”. *Neurosci. Lett.* 649 176–180. 10.1016/j.neulet.2016.07.004 27394687

[B69] Sloshower J. Guss J. Krause R. Wallace R. M. Williams M. T. Reed S. (2020). Psilocybin-assisted therapy of major depressive disorder using acceptance and commitment therapy as a therapeutic frame. *J. Contextual Behav. Sci.* 15 12–19. 10.1016/j.jcbs.2019.11.002

[B70] Spolidoro M. Sale A. Berardi N. Maffei L. (2008). Plasticity in the adult brain: lessons from the visual system. *Exp. Brain Res.* 192 335–341. 10.1007/S00221-008-1509-3 18668231

[B71] Vetencourt J. F. M. Sale A. Viegi A. Baroncelli L. de Pasquale R. O’Leary O. F. (2008). The antidepressant fluoxetine restores plasticity in the adult visual cortex. *Science* 320 385–388. 10.1126/science.1150516 18420937

[B72] Vollenweider F. X. Preller K. H. (2020). Psychedelic drugs: neurobiology and potential for treatment of psychiatric disorders. *Nat. Rev. Neurosci.* 21 611–624. 10.1038/s41583-020-0367-2 32929261

[B73] Wagner M. T. Mithoefer M. C. Mithoefer A. T. MacAulay R. K. Jerome L. Yazar-Klosinski B. (2017). Therapeutic effect of increased openness: investigating mechanism of action in MDMA-assisted psychotherapy. *J. Psychopharmacol.* 31 967–974. 10.1177/0269881117711712 28635375 PMC5544120

[B74] Wiesel T. N. (1982). Postnatal development of the visual cortex and the influence of environment. *Nature* 299 583–591. 10.1038/299583a0 6811951

[B75] Woolley S. M. N. Rubel E. W. (2002). Vocal memory and learning in adult bengalese finches with regenerated hair cells. *J. Neurosci.* 22 7774–7787. 10.1523/JNEUROSCI.22-17-07774.2002 12196601 PMC6758009

[B76] Yaden D. B. Griffiths R. R. (2021). The subjective effects of psychedelics are necessary for their enduring therapeutic effects. *ACS Pharmacol. Transl. Sci.* 4 568–572. 10.1021/acsptsci.0c00194 33861219 PMC8033615

[B77] Zeiler S. R. Gibson E. M. Hoesch R. E. Li M. Y. Worley P. F. O’Brien R. J. (2013). Medial premotor cortex shows a reduction in inhibitory markers and mediates recovery in a mouse model of focal stroke. *Stroke* 44 483–489. 10.1161/STROKEAHA.112.676940 23321442 PMC4086919

